# The first aphasia screening test in Hungarian: A preliminary study on validity and diagnostic accuracy

**DOI:** 10.1371/journal.pone.0290153

**Published:** 2023-08-17

**Authors:** Lilla Zakariás, Ágnes Lukács

**Affiliations:** 1 Bárczi Gusztáv Faculty of Special Needs Education, Eötvös Loránd University, Budapest, Hungary; 2 Faculty of Humanities, Eötvös Loránd University, Budapest, Hungary; 3 National Institute of Locomotor Diseases and Disabilities/National Institute for Medical Rehabilitation, Budapest, Hungary; 4 Department of Cognitive Science, Budapest University of Technology and Economics, Budapest, Hungary; 5 MTA-BME Momentum Language Acquisition Research Group, Eötvös Loránd Research Network (ELKH), Budapest, Hungary; Drexel University, UNITED STATES

## Abstract

The Hungarian Aphasia Screening Test (HAST) is a newly developed diagnostic tool for detecting post-stroke aphasia in clinical settings, and for differentiating between stroke patients with and without aphasia. The HAST was developed by our team and has not been published in English yet. In Hungarian, to date, there is no aphasia screening test with reported psychometric properties available. This study aims to present the main characteristics of the HAST and to evaluate its validity, internal consistency, and diagnostic accuracy. The HAST comprises five subtests (maximum score: 20) and takes 5–10 minutes to administer. We administered the HAST to 40 stroke patients with aphasia, 26 stroke patients without aphasia, and 51 healthy control participants to evaluate the test’s construct validity, convergent validity, and internal consistency, as well as its sensitivity and specificity. We used the Western Aphasia Battery (WAB) as a reference test. With a cut-off score of 17, the HAST showed high diagnostic accuracy (sensitivity: 92.5%, specificity: 88.5%). Its construct validity was good; we identified one component in the test, and moderate-to-strong positive correlations across most of its subtests (mean Spearman *r* = 0.56). Convergent validity of the HAST was satisfying, reflected by the moderate-to-strong positive correlations between subtests of the HAST and subtests of the WAB (Spearman *r* = 0.50–0.86). The correlation between the HAST total score and the WAB aphasia quotient was high (Spearman *r* = 0.86). Despite the small number of items within tasks, all subtests showed acceptable internal consistency (mean Cronbach’s *α* = 0.74). Our preliminary results suggest that the HAST is a valid, accurate, and clinically feasible test to detect post-stroke aphasia and to identify patients who require a more detailed assessment of their language skills. In addition, it reliably identifies not only the presence but also the severity of aphasia, thus, it might be a good candidate for monitoring patient progress.

## Introduction

Hungarian is spoken by 13 to 14 million people [1]; by 9,690,000 people in Hungary [2] and, as a minority language, by around three million people in the neighbouring countries, namely Slovakia, Ukraine, Romania, Serbia, Croatia, Slovenia, and Austria [1].

There are around 40,000 incident strokes and 28,000 stroke survivors each year in Hungary [3]. Around one-third of these patients present with an acquired language disorder due to stroke, namely aphasia [4, 5]. Aphasia has a negative impact on quality of life both personally and socially [6, 7]. It may negatively impact employment, education, leisure activities, finances, personal relationships, and identity of people with aphasia [8]. Families experience negative consequences of aphasia, including increased stress, more responsibilities in domestic life, decreased time for leisure, and withdrawal from social life [9]. Since aphasia significantly affects the individual, their families, and communities, its early recognition and treatment are vital [6].

The main goals of language assessment in the acute phase of stroke are to identify aphasia and determine its severity, to monitor changes in the early stage of recovery, and to facilitate the early referral to the speech-language pathologist (SLP) for further examination and early treatment of aphasia [10, 11]. In order to achieve these goals, assessment tools that can be administered in a few minutes, even at bedside, can prove to be pivotal. Ideally, such screening tests do not require specific competencies of SLPs, allowing for a use also by non-SLP health care professionals such as neurologists, psychologists, neuropsychologists, nurses, physical and occupational therapists etc. [10, 12]. Although screening tests are primarily used in the acute care of stroke, they can be useful in any clinical setting allowing for quick test administration and diagnostics. These typically include follow-up assessments to monitor change over time, assess maintenance of language skills, and decide about referral to SLPs. Some of these are often performed only by physicians in later stages of stroke care in Hungary with little time available.

El Hachioui and colleagues [10] identified eight aphasia screening tests that had been designed to determine the presence and/or severity of aphasia, had been validated in stroke patients *with* and *without* aphasia, and had data on their diagnostic accuracy (i.e., sensitivity and specificity) reported. These tests include the Frenchay Aphasia Screening Test [FAST; 13–15] and the Sheffield Screening Test [13] in English, the ScreeLing in Dutch [16], the Language Screening Test (LAST) in French [12], the Mobile Aphasia Screening Test (MAST) and the Semantic Verbal Fluency (SVF) in Korean [17 and 18, respectively], the Mississippi Aphasia Screening Test (also abbreviated as MAST) in Czech [19] and Spanish [20], and the Ullevaal Aphasia Screening test (UAS) in Norwegian [21].

To date, there is only one standardized aphasia test with reported psychometric properties available in Hungarian. The Comprehensive Aphasia Test-Hungarian [CAT-H; 22, 23] is based on the English Comprehensive Aphasia Test (CAT) developed by Swinburn, Porter, and Howard in 2004. The CAT was designed to assess language performance comprehensively and to screen for associated cognitive deficits in post-stroke aphasia [24]. The test is designed to be used by SLPs and it is typically completed in two sessions (90–120 minutes), thus its use is recommended once the patient is medically stable [24].

According to a recent systematic review on post-stroke aphasia rehabilitation, due to the high incidence of communication disorders in stroke, each stroke patient should be screened for communication deficits using a screening tool that is valid and reliable [25]. Despite the necessity of aphasia screening tests in clinical practice, in Hungarian, to date, there has not been any screening test with established psychometric properties to assess stroke patients. Therefore, we developed the Hungarian Aphasia Screening Test (HAST) for differentiating stroke patients with and without aphasia, and in the current preliminary study, we investigated the psychometric properties and the diagnostic accuracy of the HAST. During the development and validation process, as well as the data analysis, we considered several methodological recommendations and guidelines [e.g., 10, 24, 26, 27].

### Development and main characteristics of the HAST

Task and stimulus selection was based on the French LAST [12] and the English FAST [14], as well as aphasia batteries, including the CAT-H [22, 23] and the Hungarian version of the Western Aphasia Battery [WAB; 28]. Stimuli in the HAST were controlled for a number of psycholinguistic and linguistic variables known to affect language performance of people with aphasia (PWA). These included word frequency, imageability, animacy, word and sentence length, syntactic complexity, phonological and semantic relatedness, discriminability, and name agreement (relevant only for subtests involving visual stimuli) [16, 24, 29]. The development of the HAST drew upon extensive experience with the Hungarian adaptation and standardization of the CAT, a detailed battery of language abilities specifically designed for individuals with post-stroke aphasia [22, 23]. Development of the HAST included the following steps: (1) selection of tasks and verbal stimuli; (2) development of the test sheet, administration and scoring protocol; (3) construction of visual material (i.e., black and white line drawings); (4) piloting the HAST in a small group of PWA (N = 4); and (5) modification of test items and test sheet based on the pilot study, and finalizing the HAST. Main characteristics of the test are the following: (1) it assesses only spoken (expressive and receptive) language; (2) it is controlled for psycholinguistic and linguistic variables known to affect language performance in post-stroke aphasia; (3) it includes only simple visual material; and (4) it is brief (about 5–10 minutes) and easy to administer and score, thus, also suitable for use at bedside. The HAST is administered on a two-page test sheet, using a three-page visual material (see S1 and S2 Files).

The aim of the current study was to examine some of the psychometric properties and the diagnostic accuracy of the HAST in post-stroke aphasia. More specifically, we aimed to (1) compare the performance of Hungarian-speaking stroke patients with and without aphasia; (2) evaluate construct and convergent validity as well as internal consistency, item discriminability, and item difficulty of the HAST, and (3) evaluate diagnostic accuracy, i.e., sensitivity and specificity, of the HAST.

## Methods

### Procedures, stimuli, and scoring in the HAST

The HAST consists of five subtests: *Word comprehension*, *Sentence comprehension*, *Repetition*, *Naming*, and *Word fluency*. Each subtest, except *Word fluency*, includes four items. *Word fluency* comprises two tasks: category and letter fluency.

Subtest 1, *Word comprehension* is an oral word-picture matching task with semantic, phonological, and visual distractors. An array of eight black and white line drawings is presented, and the patient’s task is to select the picture matching the word read aloud by the examiner. Because word-picture matching tasks are often considered to be easy for people with aphasia, we aimed to make the task as difficult (and sensitive) as possible. Therefore, we selected short, i.e., one- or two-syllable, target words with low frequency. Low frequency words are more difficult to process than high frequency words, and, typically, short words are more difficult to process than long words [30]. The maximum score is 4 in this task.

Subtest 2, *Sentence comprehension* is an oral sentence-picture matching task (item 1–3) including semantically reversible sentences (i.e., an object-verb-subject sentence, a centre-embedded subject and a centre-embedded object relative clause). An array of four black and white line drawings is presented, and the patient’s task is to point to the line drawing representing the sentence read aloud by the examiner. For each item, there are three—either lexical or grammatical—distractor pictures and one target picture. The subtest also comprises a three-element instruction for the patient to follow (‘Point to the ceiling, then touch your nose, then your left ear’, item 4). The maximum score is 4 in this task.

Subtest 3, *Repetition* includes a four-syllable real word with low frequency and low imageability (item 1), a three-syllable nonword (item 2), a two-word phrase including a numeral (item 3), and a long sentence consisting of 24 syllables and six content words (item 4). Patients are asked to repeat the word, phrase, or sentence heard as accurately as they can. The maximum score is 4 in this task.

Subtest 4, *Naming* is a picture naming task including four black and white line drawings. Target words are three-syllable nouns with low frequency that are either inanimate (item 1 and 3) or animate (item 2 and 4). Name agreement (i.e., the consistency with which the pictures generated the target words) was tested by examining responses of the healthy control participants taking part in the study (N = 51). All pictures showed high name agreement (mean name agreement = 98.5%, SD = 1.9, range = 96–100%). The maximum score is 4 in this task.

Subtest 5, *Word fluency* consists of a category and a letter fluency task. In the category fluency task, patients are required to say out loud as many items belonging to the category “fruits” as possible in 30 seconds, while in the letter fluency task they are asked to say out loud as many words beginning with the letter “M” as possible during the same time interval. The number of correct words in the two tasks is transformed to a score between 0 and 4 in order to match the five-point scale of the other subtests (for details on scoring, see below).

Examiners were provided with a written administration and scoring protocol. In Subtest 1–4, scoring instructions were as follows: score 1 for an accurate, prompt response, as well as for a correct response after repetition of the target by the examiner (if requested, relevant only in Subtest 1–3) and/or self-correction; score 0 for an incorrect response as well as for a correct response after a semantic or phonemic cue provided by the examiner (relevant only in Subtest 4).

In Subtest 5 (*Word fluency*), examiners were instructed to sum the correct responses in the two subtasks resulting in a raw score. During the analyses, we transformed the raw scores as follows: 0 word: score 0; 1–4 words: score 1; 5–8 words: score 2; 9–15 words: score 3; 16 or more words: score 4. We used this transformation to put scores from the different subtests (i.e., *Word fluency* vs. Subtest 1–4) onto a common scale, so that each subtest contributes to the final (total) score with equal weight (for details of this transformation, see the Results). The maximum score is 20 (i.e., 5 x 4) in the test.

### Participants

We recruited 74 individuals with a medical diagnosis of stroke from three hospitals. Patients with additional neurological disorders (dementia, N = 2), psychological/psychiatric disorders (major depression, alcohol addiction, N = 2), and moderate-to-severe premorbid visual (N = 1) and auditory (N = 2) problems were excluded. One patient dropped out from the study because of COVID-19 infection. Thus, a total of 66 stroke patients participated in the study. This sample size is comparable to that of prior reports of validation of aphasia screening tests in other languages [e.g., 10, 31]. Based on the WAB, they were assigned either to the aphasia group (N = 40, WAB aphasia quotient [AQ] below 94) or to the non-aphasia group (henceforth, stroke group, N = 26, WAB-AQ > 94). The mean WAB-AQ was 60.95 for the aphasia group (SD = 27.21, range = 4.9–93.1) and 98.7 for the stroke group (SD = 1.44, range = 95.3–100). PWA presented with a range of aphasia types (5 global, 7 Broca’s, 7 transcortical motor, 2 Wernicke’s, 2 conduction, and 17 anomic). Neurogenic motor speech disorders (i.e., apraxia of speech, dysarthria) or other motor disorders due to the primary etiology (e.g., dysphagia, different types of apraxia) were not excluding factors. Demographic, medical, and clinical characteristics of the patients are summarized in Table 1.

**Table 1:** Demographic and clinical characteristics of the aphasia, stroke, and control group.

	Aphasia (N = 40)	Stroke (N = 26)	Control (N = 51)
Age (years)			
Mean (SD, range)	62.2 (14, 33–81)	63.6 (12.7, 36–82)	60.5 (12.8, 33–84)
Sex (%)			
Female	65	50	45.1
Male	35	50	54.9
Education level (%)			
8 years	7.5	7.7	15.7
11 years	30	26.9	11.8
12 years	32.5	23.1	25.5
15–17 years	27.5	42.3	45.1
Unknown	2.5	0	0
Handedness (%)			
Right	95	92.3	94.1
Left	2.5	3.85	0
Forced right	2.5	0	5.9
Mixed	0	3.85	0
Type of stroke (%)			
Infarct	62.5	84.6	
Haemorrhage	35	15.4	
Unknown cerebrovascular	2.5	0	
Lesion location (%)			
Left	77.5	57.7	
Right	5	26.9	
Bilateral	7.5	0	
Cerebellum/brainstem	10	15.4	
Post-onset (days)			
Median (SD, range)	69 (1155, 6–7082)	39.5 (2247.8, 5–11390)	
Phase of stroke (%)			
Acute (0–14 days)	7.5	19.2	
Sub-acute (15–180 days)	67.5	69.2	
Chronic (≥181 days)	25	11.6	
WAB-AQ			
Mean (SD, range)	61 (27.2, 4.9–93.1)	98.7 (1.4, 95.3–100)	
Other neurogenic communication disorders (%)			
Dysarthria	5	23.1	
Apraxia of speech	12.5	0	
Dysphagia	5	23.1	
Visual perceptual deficits	7.5	7.7	
Auditory perceptual deficits	2.5	0	

N = number of participants. Visual and auditory perceptual deficits comprise different types of anopia and visual/auditory neglect.

In addition, we recruited a group of 51 healthy participants (henceforth, healthy control group). Exclusion criteria were any neurological (e.g., dementia, epilepsy, Parkinson disease, multiple sclerosis) and psychological/psychiatric (e.g., alcohol and drug addiction, depression) disorders, as well as moderate-to-severe premorbid visual and auditory problems. All participants were native speakers of Hungarian. The three groups were matched on sex (Chi-square test, *X*^*2*^(2, N = 117) = 1.66, *p* = 0.44), age (Kruskal–Wallis test, *H*(2, N = 117) = 1.31, *p* = 0.52), and education (Chi-square test, *X*^*2*^(6, N = 116) = 8.17, *p* = 0.23). The aphasia and the stroke groups were also matched on post-onset (Wilcoxon’s rank sum test, *W* = 609, *p* = 0.25) and type of stroke (Chi-square test, *X*^*2*^(2, N = 66) = 3.95, *p* = 0.14). However, the two groups differed significantly in lesion localization (Chi-square test, *X*^*2*^(3, N = 66) = 8.77, *p* = 0.03, for details see Table 1).

Patients were recruited and tested at three clinics in Hungary: National Institute for Medical Rehabilitation (Budapest); Szent Imre University Teaching Hospital (Budapest); a clinic whose name is withheld due to a confidentiality agreement. One of the clinics is a national institute providing care for people from all over the country, while the other two are local hospitals. Altogether, one neurology ward (providing acute care) and five rehabilitation wards (providing subacute and chronic care) were involved in the study. The research was approved by the Local Research Ethics Committees (IKEB/RKEB) and the Medical Research Council (TUKEB). Participants were informed orally and in writing by the examiner about the purpose and procedures of the study and gave informed consent before participating in the study. Healthy control participants were recruited at the same clinics as patients and online via Facebook.

### Design and procedure

Administration of the HAST took from five to ten minutes for all participants. For each participant, the examiner filled out a questionnaire on demographic, medical, and clinical information. As a reference test, patients also completed the WAB [28]. WAB scores were used (1) to assign patients to the aphasia or the stroke group and (2) to assess convergent validity of the HAST. The order of administration of the HAST and the WAB (i.e., the screening and the reference test, respectively) was random across patients and the interval between administering the two tests ranged from 0 to 105 days (mean = 6.47 days, median = 1 day, SD = 16.41 days). Data collection was completed in two waves: First healthy control participants (i.e., wave 1 comprising four months), then patients (i.e., wave 2 comprising 8 months) were recruited and tested.

HAST data was collected by four SLPs (i.e., CSM, FJ, SK, and LZ [first author of the manuscript]). The WAB was administered by six SLPs (i.e., including three also collecting the HAST data, and in addition, EB, LB, and NO). HAST and WAB data was collected by different SLPs for 15% of the patients (10/66). In these cases, SLPs administering the HAST were blind to the WAB results, and vice versa. In half of the remaining cases (i.e., when the same SLP administered the HAST and the WAB), the HAST was administered first, thus, SLPs were blind to the WAB results and diagnosis.

Patients were tested in-person, whereas healthy control participants were tested in-person (N = 33) as well as online (N = 18). Due to the COVID-19 pandemic, during face-to-face testing, participants and SLPs were wearing a mouth-nose mask. During online testing, participants and SLPs did not wear a mask. To validate the online testing format, we performed a study including 10 healthy control participants (5 women; mean age = 62.78 years, SD = 10.59 years, range = 47–80 years). With each participant, an SLP (CSM or LZ) administered the HAST in-person as well as online (via Microsoft Teams); the mean time interval between the first and second test administration was 5.44 days (SD = 0.73 days, range = 4–6 days) and the order of administration of the in-person and online testing was randomized. In Subtest 1 and 5, participant scores were identical across the two testing formats. In each of Subtest 2–4, the scores differed across the two testing formats for one participant only. Because we observed excellent absolute agreement between the two testing formats, we pooled the data collected in-person and online from healthy control participants (N = 51).

### Data analysis

To compare the performance in the three participant groups, we used the Kruskal–Wallis test with the post hoc Dunnett test [32]. To investigate the factor structure of the HAST (i.e., construct validity), we performed principal components analysis [PCA; 32]. The selection of PCA was based on the recommendation by Field et al. [32] who noted that PCA is a psychometrically sound procedure to identify underlying dimensions of a data set, and it is preferred over exploratory factor analysis because of its conceptual simplicity. Factor loadings above 0.7 were considered statistically meaningful [32, 33]. To investigate the associations across subtests of the HAST (i.e., another measure of construct validity), and between subtests of the HAST and subtests of the WAB (i.e., convergent validity), we calculated Spearman rank correlations [32]. To assess homogeneity and reliability of items within subtests (i.e., internal consistency and item discriminability), we computed (1) Cronbach’s alpha, (2) corrected item to total correlation coefficients, and (3) corrected average item to item correlations coefficients [32]. Cronbach’s alpha values between 0.7 and 0.9 were considered strong [34] and correlation coefficients above 0.3 were considered good [35]. To assess diagnostic accuracy of the HAST, we (1) calculated the optimal cut-off for the total score, applying the Liu index method [36], (2) performed receiver operating characteristics (ROC) analysis to calculate area under ROC curve [AUC; 37], and (3) conducted flexible discriminant analysis [38]. All statistical analyses were implemented in R, version 1.2.1335 [39].

## Results

First, we screened the data for missing values. There was no missing data in any of the subtests of the HAST. Second, we transformed the raw scores in *Word fluency* (i.e., the sum of the correct responses in category and letter fluency) to a scale of 0–4. Transformed scores aimed to make the results of subtests with different score ranges comparable and to preserve (1) the pattern of differences in the raw scores between the stroke and the aphasia group, and (2) the pattern of individual differences in the raw scores within the aphasia group in *Word fluency*. Therefore, the intervals of the raw scores were defined based on the performance of the aphasia and the stroke group, as well as the patterns of differences between the raw scores in the two groups. The transformed score of 4 (i.e., raw score of ≥16) was reached by 42% (N = 11) of the stroke group and by 3% (N = 1) of the aphasia group. The transformed score of 3 (i.e., raw score of 9–15) was reached by 54% (N = 14) of the stroke group and by 26% (N = 9) of the aphasia group. The transformed score of 2 (i.e., raw score of 5–8) was reached by 4% (N = 1) of the stroke group and by 26% (N = 9) of the aphasia group. In the stroke group, no one reached the transformed score of 1 or 0 (i.e., raw score of 1–4 and raw score of 0, respectively). In the aphasia group, 20% (N = 7) and 26% (N = 9) of participants scored 1 and 0, respectively.

Descriptive statistics (mean, median, SD, range, skewness, and kurtosis) of the subtests are provided in Table 2 for all groups. We used the Shapiro–Wilk test to test the normality of the data distribution in each subtest (see Table 2). In the aphasia group, the Shapiro–Wilk test was significant in all subtests (*p* < 0.001) but not for the total score (*W* = 0.96, *p* = 0.23), indicating that scores in the subtests were not normally distributed. Similarly, in the stroke and the control group, the Shapiro–Wilk test was significant in all subtests as well as in the total score (*p* < 0.001).

**Table 2:** Descriptive statistics and results of the Shapiro–Wilk test of normality in subtests of the HAST for the aphasia (N = 40), stroke (N = 26), and healthy control (N = 51) group.

Group	Subtest	Mean	SD	Median	Min	Max	Skewness	Kurtosis	*W*-statistic	*p*-value
Aphasia	Word comprehension	3.25	1.21	4.00	0.00	4.00	-1.39	0.66	0.68	< 0.001
	Sentence comprehension	1.40	1.28	1.00	0.00	4.00	0.69	-0.63	0.86	< 0.001
	Repetition	2.28	1.41	2.50	0.00	4.00	-0.43	-1.11	0.87	< 0.001
	Naming	2.08	1.64	2.00	0.00	4.00	-0.08	-1.68	0.83	< 0.001
	Word fluency	1.73	1.13	2.00	0.00	4.00	-0.09	-1.16	0.89	< 0.001
	Total score	10.73	5.40	12.00	0.00	20.00	-0.27	-1.04	0.96	0.23
Stroke	Word comprehension	3.85	0.46	4.00	2.00	4.00	-2.86	7.49	0.38	< 0.001
	Sentence comprehension	3.65	0.69	4.00	2.00	4.00	-1.59	0.96	0.55	< 0.001
	Repetition	3.92	0.27	4.00	3.00	4.00	-2.99	7.25	0.30	< 0.001
	Naming	3.81	0.40	4.00	3.00	4.00	-1.47	0.18	0.48	< 0.001
	Word fluency	3.54	0.51	4.00	3.00	4.00	-0.15	-2.05	0.64	< 0.001
	Total score	18.77	1.48	19.00	13.00	20.00	-2.26	6.25	0.71	< 0.001
Control	Word comprehension	4.00	0.00	4.00	4.00	4.00	-	-	0.47	< 0.001
	Sentence comprehension	3.76	0.55	4.00	1.00	4.00	-2.89	10.12	0.30	< 0.001
	Repetition	3.92	0.27	4.00	3.00	4.00	-3.04	7.41	0.25	< 0.001
	Naming	3.94	0.24	4.00	3.00	4.00	-3.64	11.48	0.44	< 0.001
	Word fluency	3.84	0.37	4.00	3.00	4.00	-1.83	1.38	0.67	< 0.001
	Total score	19.47	0.83	20.00	17.00	20.00	-1.43	1.11	0.47	< 0.001

N = number of participants; SD = standard deviation. The maximum score in all subtests is 4. The maximum total score is 20.

Values of skewness and kurtosis greater than +1 or lower than –1 [40] indicate a non-normal distribution. Based on Hair et al. [40], non-normality was observed in all subtests for all groups except in *Sentence comprehension* in the aphasia group.

Next, we examined the relationship between the demographic factors (i.e., age, sex, and education) and the HAST total score for each group separately. In the aphasia group, the HAST total score did not show a significant relationship with age (Spearman’s correlation, *r* = –0.10, *p* = 0.52), sex (Wilcoxon’s rank sum test, *W* = 220, *p* = 0.29), or education (Spearman’s correlation, *r* = 0.09, *p* = 0.57). Similarly, in the stroke group, the HAST total score did not show a significant relationship with age (Spearman’s correlation, *r* = –0.28, *p* = 0.17), sex (Wilcoxon’s rank sum test, *W* = 86, *p* = 0.96), or education (Spearman’s correlation, *r* = 0.29, *p* = 0.15). Finally, in the healthy control group, there was no significant relationship between age (Spearman’s correlation, *r* = –0.21, *p* = 0.14), sex (Wilcoxon’s rank sum test, *W* = 259.5, *p* = 0.17), and the HAST total score. However, education showed a significant relationship with the HAST total score (Spearman’s correlation, *r* = 0.55, *p* < 0.001).

### Comparing performance in the aphasia group to performance in the stroke and the control group in subtests of the HAST

Because the distribution of scores on subtests was non-normal, we used the non-parametric Kruskal–Wallis test to compare the performance of the three groups [32]. To control for false positives due to multiple comparisons, we divided the conventional alpha level by the number of comparisons (i.e., 5 HAST subtests + 1 HAST total score = 6), setting the alpha value to 0.05/6 = 0.008. Table 3 summarizes the results of the Kruskal–Wallis tests. These tests showed a significant difference between the three groups in all subtests as well as in the total score. Post hoc Dunnett tests showed that the aphasia group performed significantly lower than the stroke and the control group in all subtests as well as in the total score (adjusted *p* < 0.01 for all comparisons). In addition, performance of the stroke group did not differ significantly from that of the control group in any of the subtests (*p* > 0.05 for all comparisons). *P*-values in the Dunnett test were adjusted using the Benjamini-Hochberg method to control for the false discovery rate [41]. Results of the Dunnet test are provided in S1 Table.

**Table 3:** Results of the Kruskal–Wallis test comparing performance of the aphasia (N = 40), the stroke (N = 26), and the control (N = 51) group in subtests of the HAST.

Subtest	Possible max	Aphasia		Stroke		Control		Statistics	
		Mean	SD	Mean	SD	Mean	SD	*H*	*p*
Word comprehension	4	3.25	1.21	3.85	0.46	4	0	22.58	< 0.001
Sentence comprehension	4	1.40	1.28	3.65	0.69	3.76	0.55	66.86	< 0.001
Repetition	4	2.28	1.41	3.92	0.27	3.92	0.27	62.72	< 0.001
Naming	4	2.08	1.64	3.81	0.40	3.94	0.24	50.77	< 0.001
Word fluency	4	1.73	1.13	3.54	0.51	3.84	0.37	76.66	< 0.001
Total score	20	10.73	5.40	18.77	1.48	19.47	0.83	74.72	< 0.001

N = number of participants; SD = standard deviation.

### Validity of the HAST

To explore the relationship between subtests of the HAST (i.e., a measure of construct validity), we conducted Spearman rank correlations in the aphasia group. Again, to control for false positives due to multiple comparisons, we divided the conventional alpha level by the number of comparisons (i.e., for the five subtests, 4 + 3 + 2 + 1 = 10), setting the alpha value to 0.05/10 = 0.005. Table 4 summarizes the results of the Spearman rank test. Except for *Sentence comprehension*, all subtests showed moderate-to-strong significant positive correlations with each other (mean *r* = 0.56). The correlation was highest for *Repetition* and *Naming* (*r* = 0.78), and lowest for *Word comprehension* and *Sentence comprehension* (*r* = 0.3). *Sentence comprehension* was significantly correlated only with *Naming* (*r* = 0.65) and *Word fluency* (*r* = 0.47).

**Table 4:** Spearman rank correlations across subtests of the HAST in the aphasia group (N = 40).

HAST subtest	Word comprehension	Sentence comprehension	Repetition	Naming	Word fluency
Word comprehension	1	0.3	0.54*	0.48*	0.67*
Sentence comprehension		1	0.41	0.65*	0.47*
Repetition			1	0.78*	0.63*
Naming				1	0.66*
Word fluency					1

N = number of participants;

* statistically significant correlations (*p* < 0.005).

To explore the factor structure in the HAST (i.e., to assess construct validity), we conducted a PCA on the five subtests using the “psych” package in R [42]. The Kaiser–Meyer–Olkin (KMO) measure supported the sampling adequacy for the analysis with an overall KMO = 0.75 (with KMO values between 0.70 and 0.82 generally classified as ‘good’), and all KMO values for individual subtests were above the acceptable limit of 0.50 [32]. Bartlett’s test of sphericity, *χ*^*2*^(10) = 102.045, *p* < 0.001, indicated that correlations between subtests were sufficiently large for PCA. An initial analysis was run to obtain eigenvalues for each component in the data. One component had an eigenvalue over Kaiser’s criterion of 1 and explained 65% of the variance. The scree plot also justified retaining one component. Based on the scree plot and Kaiser’s criterion on one component, we retained one component in the final analysis. With the exception of *Sentence comprehension*, all subtests loaded above 0.7 on this component (see Table 5), with coefficients ranging from 0.74 (i.e., *Word comprehension*) to 0.89 (i.e., *Naming*). The relatively low factor loading in *Sentence comprehension* (i.e., 0.68) suggests that performance in this subtest was the least strongly related to the construct underlying behaviour in the HAST [33]. Stevens [33] recommends that for a sample size of 50 a loading of 0.72 can be considered significant. Because our sample included 40 participants, we followed his recommendation and considered loadings above 0.7 statistically meaningful.

**Table 5:** Summary of principal components analysis results for the HAST (N = 40).

Subtest	Factor 1
Word comprehension	**0.74**
Sentence comprehension	0.68
Repetition	**0.84**
Naming	**0.89**
Word fluency	**0.86**
*Variance explained (%)*	*65*

*Note*. Factor loadings over 0.70 appear in bold.

To test whether HAST scores correlated with another test of closely related constructs (i.e., to assess convergent validity), we calculated Spearman rank correlation coefficients between subtests of the HAST and subtests of the WAB. Results (Table 6) showed moderate to strong positive correlations between the subtests. Again, to control for false positives due to multiple comparisons, we divided the conventional alpha level by the number of comparisons (3 for HAST *Word comprehension* + 3 for HAST *Sentence comprehension* + 2 for HAST *Repetition* + 4 for HAST *Naming* + 4 for HAST *Word fluency* + 1 for HAST total score = 17), setting the alpha value to 0.05/17 = 0.003. All correlations were statistically significant. The correlation was highest for the HAST total score and the WAB-AQ (i.e., 0.86), and lowest for HAST *Sentence comprehension* and WAB Sequential commands (i.e., 0.50) potentially due to the different formats of these tasks (i.e., sentence-to-picture matching with a choice of four in the HAST and following instructions in the WAB). Lower correlations may also be due to the small number of items (i.e., 4) in subtests of the HAST and thus, the little variance in the data.

**Table 6:** Spearman rank correlations between subtests of the HAST and subtests of the WAB in the aphasia group (N = 40).

HAST subtest	WAB							
	Auditory verbal comprehension			Repetition	Naming and word finding			WAB-AQ
	Word recognition	Sequential commands	Comprehension Total		Object naming	Word fluency	Naming Total	
Word comprehension	0.59		0.60					0.57
Sentence comprehension		0.50	0.51					0.55
Repetition				0.76				0.80
Naming					0.77	0.78	0.80	0.77
Word fluency					0.64	0.66	0.71	0.75
HAST Total score								0.86

N = number of participants; WAB = Hungarian adaptation of the Western Aphasia Battery [28]; AQ = aphasia quotient. All correlations were statistically significant (*p* < 0.001).

### Internal consistency, item discriminability, and item difficulty in subtests of the HAST

To measure homogeneity of items within subtests (i.e., to assess internal consistency), we computed the Cronbach’s alpha, using the “psych” package in R [42]. Despite the small number of items within tasks (i.e., 4), all subtests except *Sentence comprehension* showed acceptable or good reliability in the HAST [32]. Cronbach’s alpha values ranged from 0.58 for *Sentence comprehension* to 0.84 for *Naming*. Poor reliability in *Sentence comprehension* may be related to the high rate of chance-level performance among PWA in this task. See Table 7 for the Cronbach’s alpha values and estimated confidence intervals.

**Table 7:** Reliability coefficients (Cronbach’s alpha) for subtests of the HAST (N = 40).

Subtest	No. of items	*α*	95% CI	
			LCF	UCF
Word comprehension	4	0.77	0.535	0.890
Sentence comprehension	4	0.58	0.208	0.754
Repetition	4	0.76	0.607	0.856
Naming	4	0.84	0.715	0.910

*Note*. N = number of participants, *α* = Cronbach’s alpha, LCF = lower confidence limit, UCF = upper confidence limit.

In addition, as part of the item analysis, we calculated (1) corrected average item to item correlation coefficients and (2) corrected item to total correlation coefficients (i.e., item discriminability) (see S2 Table for individual item values). In all subtests except *Sentence comprehension*, corrected average item to item correlation coefficients were above 0.3 (mean *r* = 0.44). Correlation coefficients ranged from 0.26 for *Sentence comprehension* to 0.56 for *Naming*. The mean of corrected item to total correlation coefficients across subtests ranged between 0.37 and 0.67 (mean *r* = 0.55), and most of the correlation coefficients were above the desired value of 0.3 [for a guideline on item analysis and critical values, see 32, 35]. Only two items in *Sentence comprehension* showed low item reliability (corrected item to total correlation between 0.2 and 0.3). The relatively lower values for *Sentence comprehension* may reflect close to floor effects in the subtest.

Next, we calculated item difficulty, also termed item passing rates (average score for each item) based on performance of the aphasia group (see S3 Table for individual item passing rates for all subtest). The mean of item passing rates across subtests ranged from 0.35 for *Sentence comprehension* to 0.81 for *Word comprehension*. This reflects the fact that tasks targeting word comprehension are typically easier for PWA than tasks targeting syntactic comprehension or expressive language abilities. Relative to other subtests of the HAST, *Repetition* showed a wider spread in item passing rates (0.30–0.70). The narrower spread in item passing rates and the generally lower values in *Sentence comprehension* (0.28–0.40) suggest that syntactic comprehension abilities are markedly impaired in aphasia. The generally narrow spread in item passing rates in subtests of the HAST (S3 Table) reflects the fact that we aimed to create subtests of high difficulty and of high sensitivity. Overall, the item analysis indicated acceptable reliability in subtest items and an appropriate structure of the HAST, and it suggested that the test is sensitive to even mild forms of aphasia.

### Diagnostic accuracy of the HAST

To define the optimal cut-off score in the HAST, we used the Liu index method [43]. This method defines the optimal cut-off value as the score maximizing the product of sensitivity (true positive rate) and specificity (true negative rate) over all possible cut-off values [36, 43]. This method resulted in the cut-off score of 17: patients were classified as aphasic if they scored 17 or less in the HAST. Applying the cut-off of 17 identified correctly 92.5% of stroke patients with aphasia (37/40) and 88.5% of stroke patients without aphasia (23/26). This cut-off also identified correctly 96.1% of healthy control participants (49/51). PWA scoring above the cut-off score comprised two individuals with mild anomic aphasia (WAB-AQ = 89.6 and 93.1), and one participant who, albeit scored generally high (between point 8 and 10) in most subtests of the WAB, scored low (point 4) on the Speech fluency subtest, resulting in a WAB-AQ of 83 and the diagnosis of transcortical motor aphasia. The fact that the HAST does not assess speech fluency might explain the discrepancy between the two test results in case of this participant.

**Fig 1:**
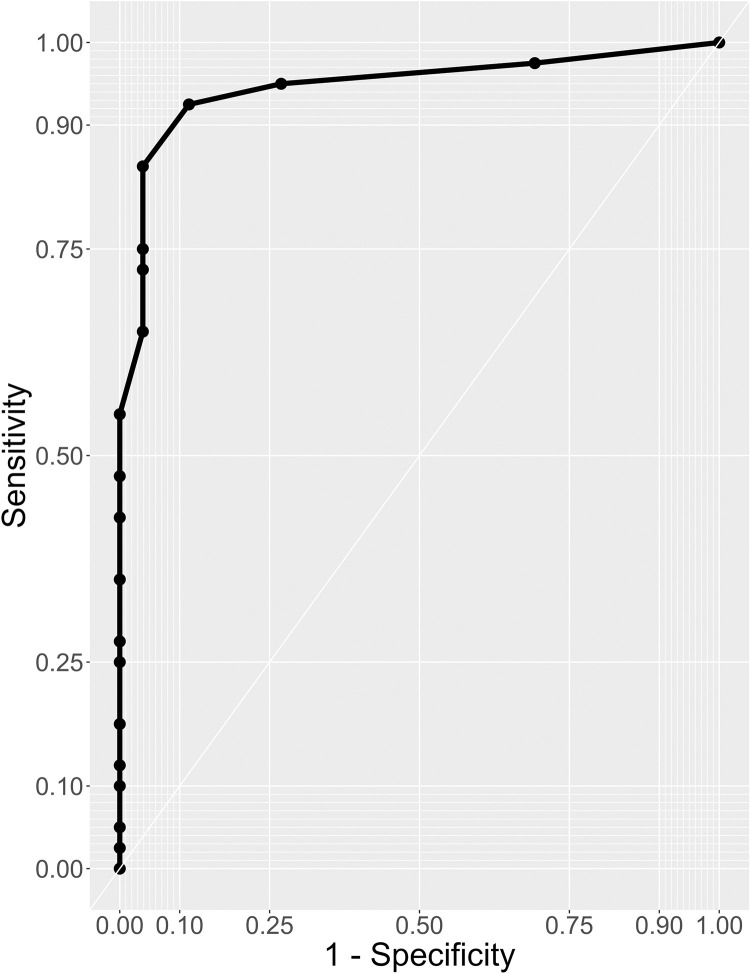
ROC curve of HAST total score. We defined the optimal cut-off score using the Liu index method, resulting in the cut-off score of 17. Applying this cut-off, sensitivity and specificity were 92.5% and 88.5%, respectively. The area under the curve (AUC) was 0.95, suggesting high diagnostic accuracy for the HAST.

We also performed receiver operating characteristics (ROC) analysis to assess the effectiveness of the HAST in differentiating between people with aphasia and people with stroke without aphasia (see Fig 1). We plotted the ROC curve in R using the “plotROC” package [44]. The discriminatory power of the HAST was determined by the area under the curve [AUC; 37]. In our sample the AUC was 0.95 (95% CI [0.89, 1.00]), indicating excellent diagnostic accuracy of the HAST (Fig 1).

Finally, we performed nonparametric flexible discriminant analysis (FDA) to investigate the effectiveness of subtest scores in differentiating between stroke patients with and without aphasia, using the “mda” package in R [45]. The FDA using all five subtest scores correctly classified 92.5% of stroke patients with aphasia (37/40) and 96.2% of stroke patients without aphasia (25/26), with largest loadings on the discrimination function of *Word fluency* (0.82) and *Sentence comprehension* (0.69).

## Discussion

The aims of the current paper were to describe a newly developed Hungarian aphasia screening test—the HAST—for assessing post-stroke aphasia, and to evaluate its construct and convergent validity, internal consistency, as well as its diagnostic accuracy. We designed the HAST to identify PWA in clinical settings, that is, to successfully differentiate between stroke patients *with* and *without* aphasia. Our study included stroke patients with and without aphasia (N = 66) as well as healthy control participants (N = 51).

The HAST is brief (around 5–10 minutes) to administer and easy to score, therefore it can also be used at bedside. Clinical feasibility of the HAST is also reflected by the fact that there was no missing data in the study; all participants with various severity of stroke and aphasia were able to complete the test. Psychometric evaluation of our results shows that the HAST has good-to-excellent psychometric properties: it is able to differentiate between stroke patients with and without aphasia, and it demonstrates acceptable internal consistency as well as good validity.

Most importantly, the HAST has high diagnostic accuracy. With a cut-off score of 17, it showed high sensitivity and specificity (92.5% and 88.5%, respectively). Only 7.5% of stroke patients (3 out of 40) who had a reference diagnosis of “aphasia” scored above the cut-off in the HAST. Two of these patients had mild anomic aphasia according to the reference test (WAB); the third patient was diagnosed as aphasic due to his/her low speech fluency score on the WAB. The fact that the HAST does not assess speech fluency might explain the discrepancy between the two test results in case of this participant. Eleven and a half percent of patients (3 out of 26) had a HAST-score equal or below the cut-off, whereas their reference diagnosis was “no aphasia”. Two of them scored 17 (i.e., equal to the cut-off); the third patient was five days post-onset—the most acute case among our group of patients. Thus, it may well be that his/her performance was the most affected by confounding variables. Indeed, this patient had a left incomplete hemianopia, which might have resulted in the low scores (i.e., point 2) in two of the subtests involving visual material in the HAST. This suggests that when interpreting the HAST results, other clinical features such as hemianopia or neglect should be considered because these could reduce the test’s specificity. It is also important to emphasize that the HAST does not replace the need for a comprehensive speech-language evaluation: anyone scoring 17 or below should be assessed comprehensively using available diagnostic tests (i.e., CAT-H or WAB in Hungarian) to identify specific areas of impairment, concomitant cognitive disorders, and communication strengths and weaknesses. In acute aphasia, we recommend readministering the HAST after a few days (ideally about 2 to 3 weeks post-onset) and performing the comprehensive speech-language assessment in case the aphasia is still present in the subacute phase.

Results of our preliminary study demonstrated good construct validity for the HAST in people with post-stroke aphasia. We identified one component in the HAST. This is in line with results of Flamand-Roze [12], also identifying a one-dimension structure for their aphasia screening test (i.e., LAST). In the HAST *Sentence comprehension* had the lowest component loading (i.e., 0.68), suggesting that performance in this subtest is the least strongly related to the construct underlying behaviour in the HAST. Although internal consistency was also the poorest in *Sentence comprehension* (Cronbach’s *α* = 0.58), the additional item analysis—corrected item to total correlation coefficients and item passing rates—indicated acceptable reliability for most items (mean *r* = 0.55) and high sensitivity of this subtest. More importantly, the fact that this subtest also showed good discrimination function between stroke patients with and without aphasia confirmed the need to include this subtest in the final version of the HAST. We consider two alternative explanations for the one-component structure in the HAST: (1) it might be driven by the strong severity-related inter-correlations between the subtests, suggesting that stroke affects language on a general level, or (2) as the subtests contain only four items (resulting in a score ranging from 0 to 4 in each subtest), variance in the subtest scores might not be large enough for the PCA to separate the constructs the subtests measure.

Moderate-to-strong inter-correlations across most of the HAST subtests also suggest that performance on one subtest is associated with performance on other subtests. An exception to this is *Sentence comprehension* which was significantly correlated only with *Naming* and *Word fluency*. Despite the moderate-to-strong inter-correlations across subtests, performance on certain subtests can be more impaired than on others in a given individual.

As shown by the moderate-to-strong positive correlations between subtests of the HAST and subtests of the WAB, the HAST has good convergent validity. All of the correlations were statistically significant, and despite the different format of some of the compared tasks, the correlations were generally high. Importantly, high correlation between the HAST total score and the WAB-AQ (i.e., 0.86) suggests that the HAST reliably identifies not only the presence but also the severity of aphasia. In sum, our preliminary results suggest that the HAST is a valid, accurate, and clinically feasible test to diagnose post-stroke aphasia.

### Limitations of the study and future directions

Among the limitations of our study, we first highlight that we did not implement consecutive patient inclusion (i.e., assessment of all patients admitted to the participating hospitals during the time of study) in the current study. Nevertheless, we aimed to recruit as representative and heterogenous sample of stroke patients as possible. Thus, when patients were referred to the SLPs taking part in the study, and patients were willing to participate, SLPs included them in the study regardless of the severity of their condition.

Second, our sample included patients with a wide range of post-onset. Because our study mainly took place at rehabilitation departments (and not in emergency and acute care settings), we did not have the possibility to include acute patients only (i.e., post-onset < 14 days). In fact, the majority of our patients was in the subacute and chronic phase of stroke. We believe that the HAST will be used in various clinical settings not only in the acute, but also in the subacute and chronic phases of stroke in Hungary, thus, we do not believe that the variance in post-onset of our patient sample is problematic for the validation study. Nevertheless, future studies implementing consecutive patient inclusion in acute care settings should inform practitioners about the validity and diagnostic accuracy of the HAST in this specific group of patients.

Third, our sample consisted of only 40 people with aphasia. It is important to note that the small sample size poses a limitation to the interpretation of the PCA results. In future studies, it will be important to investigate construct validity, specifically the factor structure of the HAST, on a larger sample using confirmatory factor analysis. This analysis could for instance compare a theoretically justified factor solution to the default one-factor solution. Future studies should also focus on evaluating the content validity of the HAST by involving a panel of experts through focus groups and interviews. This process should also be accompanied by a detailed description and documentation of the motivation behind selecting the subtests and items of the test.

The fourth limitation of our study is that the WAB, which was used as a reference test in our study, is adapted but not standardized in Hungarian, thus, it cannot be considered a “gold standard” for diagnosing PWA. The only standardized aphasia test with reported psychometric properties in Hungarian is the CAT-H. Although the CAT-H can be used for aphasia diagnosis and has good psychometric properties [22], it is a very recent test and to date, clinical practice still relies on the WAB to assess PWA in Hungary. In addition, the use of the CAT-H is recommended once the patient is medically stable, i.e., primarily in the subacute and chronic stages of stroke. For the above reasons, we used the WAB as the reference test, which might have led to misclassification of some of the stroke patients in our study. Since it has been previously shown that the scores in the CAT-H and the WAB highly correlate with each other [22], we expect that using the WAB did not have a substantial effect on our results regarding the diagnostic accuracy of the HAST. Nevertheless, future studies using the CAT-H as a reference test might provide more precise information on the diagnostic accuracy of the HAST.

Next, it is also important to note that for three PWA, the time interval between the administration of the WAB and the HAST was relatively long (i.e., 26, 39, and 105 days). At the first test administration, these PWA were in the subacute phase of stroke (i.e., 54-, 162-, and 143-days post-onset) and showed marked difficulties in both tests. The long time interval between the two tests may have weakened observed correlations in the convergent validity analysis. Nevertheless, the correlation analysis suggested good convergent validity of the HAST, thus, we believe that including these PWA in the analysis did not substantially affect our results.

Finally, we developed the HAST for use also by non-SLPs (e.g., physicians, psychologists, and nurses). However, reliable use of the HAST needs to be confirmed by future studies specifically investigating the consistency of test results across different examiners. Additionally, clinical feasibility should also be examined by calculating missing values when the test is administered by professionals other than SLPs. The consistency of test results across different measurement occasions (i.e., test–retest reliability of the HAST) also warrants future investigations. Future studies are necessary to demonstrate the test’s ability to monitor long-term changes in aphasia over time and to detect meaningful changes in the patients’ performance due to an intervention, a property termed responsiveness.

Taken together, our preliminary results suggest that the HAST is an accurate test for detecting aphasia after stroke and identifying patients who require a more detailed assessment of their language and communication skills. The high correlation between the HAST total score and the WAB-AQ, as well as the normal distribution of the HAST total score suggest that the HAST is able to detect not only the presence but also the severity of aphasia, thus, it might be a good candidate for monitoring patient progress. We believe that the HAST will be a unique resource for health care professionals and aphasia researchers in aphasia assessment and diagnostics in Hungary.

## Supporting information

S1 Text Target items of the HAST and their translations. (PDF)

S1 File Test sheet of the HAST. (PDF)

S2 File Visual material of the HAST. (PDF)

S1 Table Results of the Dunnet-test in subtests of the HAST. (DOCX)

S2 Table Corrected item to total correlations (item discriminability) and corrected average item to item correlations in the HAST subtests (N = 40). (DOCX)

S3 Table Item passing rates (item difficulty) of the HAST subtests (N = 40). (DOCX)

S1 Dataset Full dataset. (XLSX)
