# Supplementary material for: The first aphasia screening test in Hungarian: A preliminary study on validity and diagnostic accuracy
Source: PLoS One. 2023 Aug 17;18(8):e0290153. doi: 10.1371/journal.pone.0290153 (PMC10434950; doi:10.1371/journal.pone.0290153)
Supplement: S1 Text — (PDF) [file pone.0290153.s001.pdf]

The Hungarian Aphasia Screening Test: Target items with translations

1. Word comprehension

1. *labda* ‘ball’
2. *domb* ‘hill’
3. *gyík* ‘lizard’
4. *sín* ‘rails’

2. Sentence comprehension

1. *A fehér elefántot kergeti a zsiráf*	‘The white elephant is being chased by the giraffe’
2. *A zsiráf, amit az elefánt kerget, az fehér*	‘The giraffe that the elephant chases is white’
3. *Az elefánt, ami a zsiráf mögött van, az fehér*	‘The elephant that is behind the giraffe is white’
4. *Mutasson a plafonra, majd érintse meg az orrát, aztán a bal fülét!*	‘Point to the ceiling, then touch your nose, then your left ear’

3. Repetition

1. *metafora*	‘metaphor’
2. *zoréda* (nonword)	-
3. *százötvenhat kilométer*	‘one hundred and fifty-six kilometers’
4. *A három unokatestvérnek annyira tetszett a film, hogy még kétszer megnézték.*	‘The three cousins liked the movie so much that they watched it twice’

4. Naming

1. *korona* ‘crown’
2. *denevér* ‘bat’
3. *piramis* ‘pyramid’
4. *kenguru* ‘kangaroo’

5. Word fluency

- Fruits
- words starting with ‘m’
