# Supplementary material for: The first aphasia screening test in Hungarian: A preliminary study on validity and diagnostic accuracy
Source: PLoS One. 2023 Aug 17;18(8):e0290153. doi: 10.1371/journal.pone.0290153 (PMC10434950; doi:10.1371/journal.pone.0290153)
Supplement: S1 File — (PDF) [file pone.0290153.s002.pdf]

MAGYAR AFÁZIA SZŰRŐTESZT (MASZT): VIZSGÁLATI ŰRLAP

Zakariás Lilla & Lukács Ágnes

Szükséges eszközök: KÉPANYAG az 1., 2. és 4. feladathoz; stopper vagy másodpercmutatós óra az 5. feladathoz. Ha a páciens kéri, az 1–3. feladatban a vizsgálatvezető 1-szer megismételheti a szó/mondat/célelem bemutatását.

**Páciens neve:**<br>**Születési dátum, életkor:**<br>**Lakcím (település):**	**Tesztfelvétel időpontja:**<br>**Vizsgálatvezető neve:**<br>**Vizsgálatvezető foglalkozása:**

1. SZÓÉRTÉS

Instrukció: *Egy szót fogok mondani. Mutassa meg, amit mondtam!*
A VÁLASZ oszlopban karikázza be a páciens válaszát.

	CÉLSZÓ	VÁLASZ				PONT
1	labda	gyík	paradicsom (V)	gomb	sín	0 1
		**labda**	kígyó	domb	síp	
2	domb	gyík	paradicsom	gomb (F)	sín	0 1
		labda	kígyó	**domb**	síp	
3	gyík	**gyík**	paradicsom	gomb	sín	0 1
		labda	kígyó (SZ)	domb	síp	
4	sín	gyík	paradicsom	gomb	**sín**	0 1
		labda	kígyó	domb	síp (F)	
					**PONTSZÁM**	**/4**

V: vizuális elterelő, F: fonológiai elterelő, SZ: szemantikai elterelő

2. MONDATÉRTÉS

Instrukció (1–3. próba): *Nézze meg alaposan mind a négy képet. Egy mondatot fogok mondani. Mutassa meg, amit mondtam!*
Instrukció (4. próba): *Pontosan csinálja meg, amit kérek! Először hallgassa végig a teljes mondatot, és csak utána csinálja meg, amit kértem!*
A VÁLASZ oszlopban karikázza be a páciens válaszát.

	CÉLMONDAT	VÁLASZ		PONT
1	A fehér elefántot kergeti a zsiráf	1	2	0 1
		3	**4**	
2	Az elefánt, ami a zsiráf mögött van, az fehér	1	**2**	0 1
		3	4	
3	A zsiráf, amit az elefánt kerget, az fehér	**1**	2	0 1
		3	4	
4	Mutasson a plafonra, majd érintse meg az orrát, aztán a bal fülét!			0 1
			**PONTSZÁM**	**/4**

3. ISMÉTLÉS

Instrukció: *Ebben a feladatban egy szót, egy értelmetlen szót, vagy egy mondatot fogok mondani. Pontosan, szóról-szóra ismételje meg, amit mondtam!*
Jegyezze le a páciens válaszát. Csak a teljesen pontos, hibátlan választ fogadja el.

	CÉLELEM	VÁLASZ (ÁTIRAT)	PONT
1	metafora		0 1
2	zoréda (álszó)		0 1
3	százötvenhat kilométer		0 1
4	A három unokatestvérnek annyira tetszett a film, hogy még kétszer megnézték		0 1
		**PONTSZÁM**	**/4**

4. MEGNEVEZÉS

Instrukció: *Egyenként nevezze meg a képeket, mindegyiket egy szóval! Haladjon sorban, balról-jobbra!*
Csak a pontos és segítség nélkül adott választ fogadja el. A diszartriás és enyhe beszédapraxiás hibákért (hangtorzításokért) ne vonjon le pontot, amennyiben a válasz egyébként helyes.

	CÉLSZÓ	VÁLASZ (ÁTIRAT)	PONT
1	korona		0 1
2	denevér (bőregér)		0 1
3	piramis		0 1
4	kenguru		0 1
		PONTSZÁM	/4

5. SZÓFLUENCIA

Instrukció: *A következő feladatra 30 másodperce lesz. Soroljon fel minél több szót, ami a „gyümölcs" kategóriához tartozik! Tulajdonneveket (pl. Idared), kérem, ne mondjon! Szólok, ha kezdheti. ... Most!*

GYÜMÖLCSÖK	
SZAVAK SZÁMA:	

Instrukció: *Most egy másik feladat jön. Erre is 30 másodperce lesz. Soroljon fel minél több szót, ami „m" hanggal kezdődik! Tulajdonneveket (pl. Mária), kérem, ne mondjon! Szólok, ha kezdheti. ... Most!*

„M"	
SZAVAK SZÁMA:	

Adja össze a helyes válaszok számát a két feladatban, és az így kapott értéket alakítsa át a következők szerint:

0 szó: 0 pont	1–4 szó: 1 pont	5–8 szó: 2 pont	9–15 szó: 3 pont	16 szó: 4 pont
			SZÓFLUENCIA PONTSZÁM	/4

NYELVI ÖSSZPONTSZÁM<br>(SZÓÉRTÉS + MONDATÉRTÉS + ISMÉTLÉS + MEGNEVEZÉS + SZÓFLUENCIA)	/20

0–8 pont: súlyos afázia	9–13 pont: közepesen súlyos afázia	14–17 pont: enyhe afázia	18–20 pont: nincs afázia

**Nem**: ☐ férfi ☐ nő **Kezesség**:	**Agyi történés időpontja**:
**Anyanyelv**: **Második nyelv** (opcionális):	**Etiológia**: ☐ infarktusos stroke ☐ vérzéses stroke ☐ traumás agysérülés ☐ tumor ☐ egyéb:
**Iskolai évek száma, legmagasabb végzettség**:	**Stroke esetén**: ☐ arteria cerebri media ☐ arteria cerebri anterior ☐ arteria cerebri posterior ☐ arteria basilaris/vertebralis ☐ egyéb:
**Foglalkozás**:	**Első stroke**: ☐ igen ☐ nem **Korábbi stroke időpontja:**
**Látás** (szemüveg, egyéb):	**Lokalizáció**: ☐ bal ☐ jobb ☐ kétoldali ☐ kisagy/agytörzs ☐ egyéb:
**Hallás** (hallókészülék, egyéb):	**Pontos lokalizáció**:
**Orvosi diagnózis, BNO-kód**:	
**Egyéb neurológiai vagy pszichiátriai zavar**:	
**Egyéb (beszéd)motoros zavar**: ☐ beszédapraxia ☐ diszartria ☐ diszfágia ☐ orális apraxia ☐ apraxia	
