# Supplementary figures and images for: The first aphasia screening test in Hungarian: A preliminary study on validity and diagnostic accuracy
Source: PLoS One. 2023 Aug 17;18(8):e0290153. doi: 10.1371/journal.pone.0290153 (PMC10434950; doi:10.1371/journal.pone.0290153)

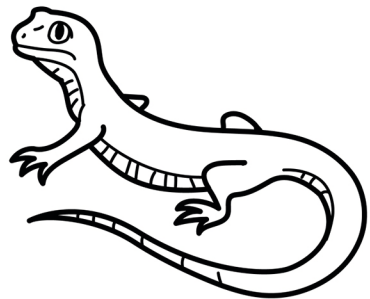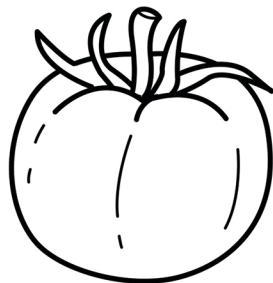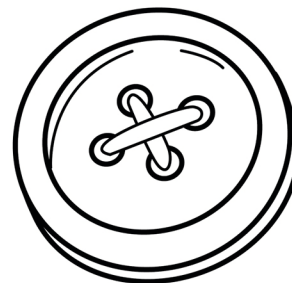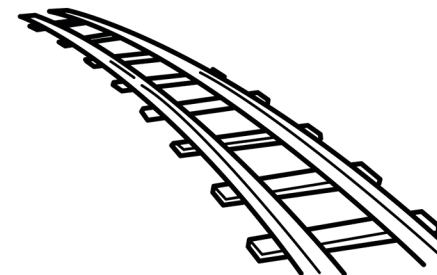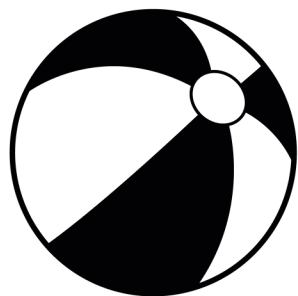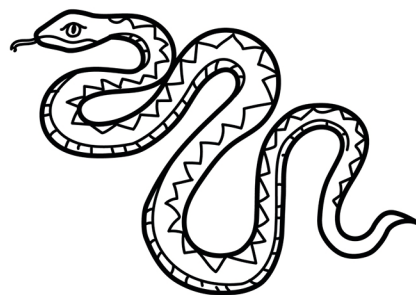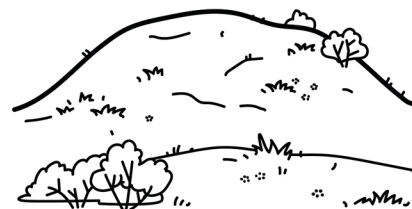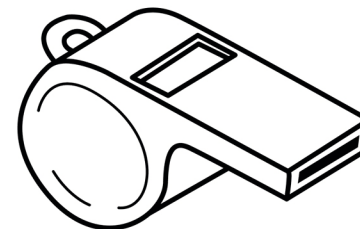

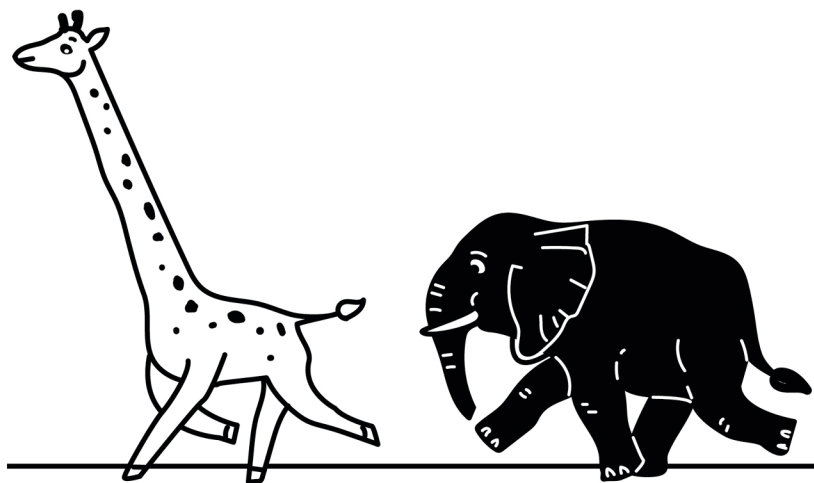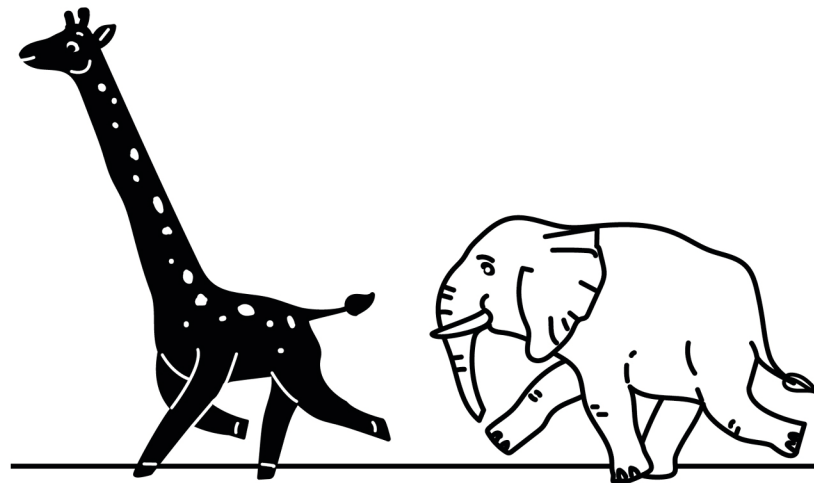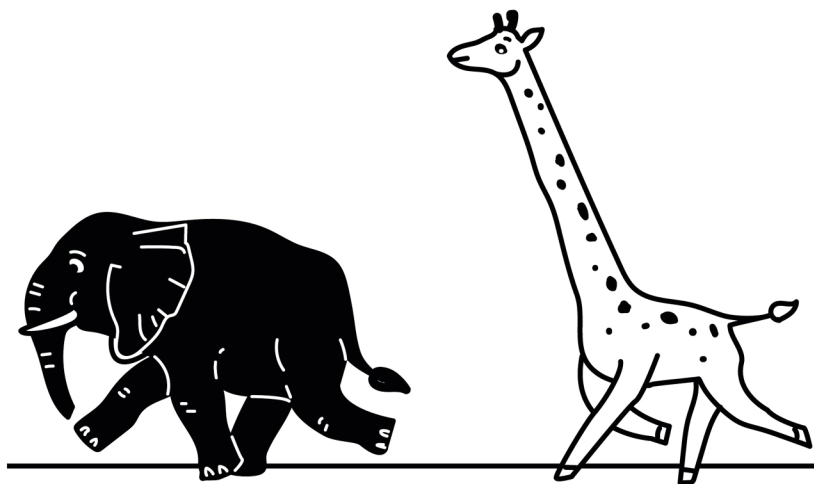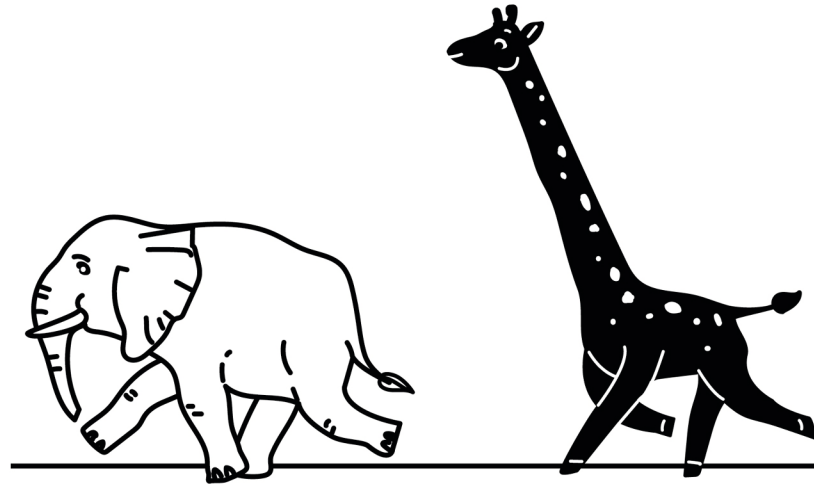

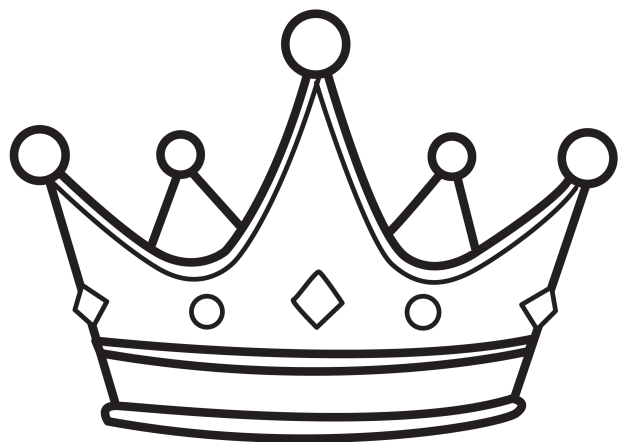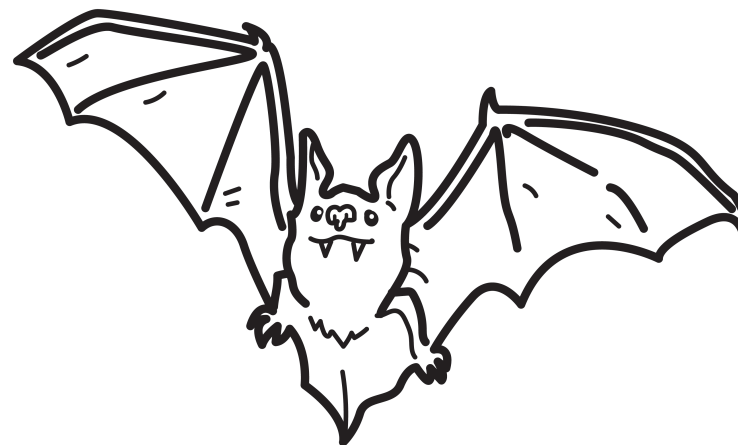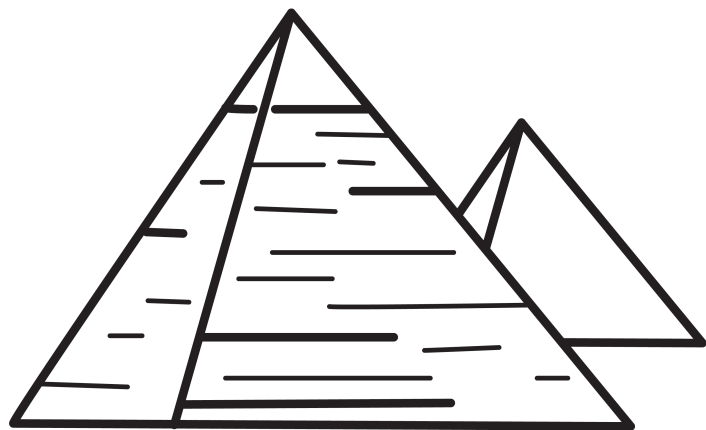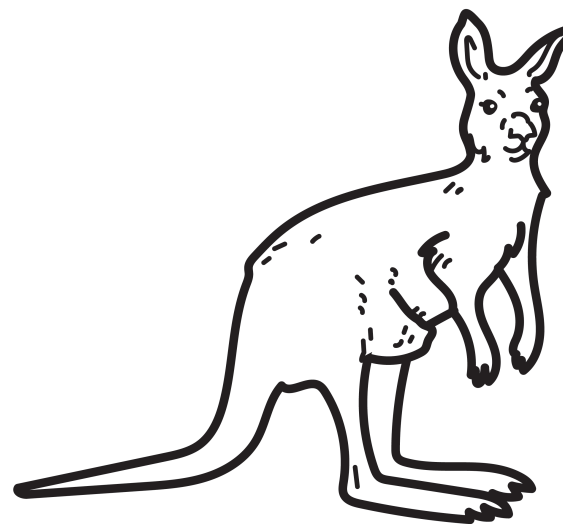

Supplement: S2 File — (PDF) [file pone.0290153.s003.pdf]
