# Supplementary material for: The first aphasia screening test in Hungarian: A preliminary study on validity and diagnostic accuracy
Source: PLoS One. 2023 Aug 17;18(8):e0290153. doi: 10.1371/journal.pone.0290153 (PMC10434950; doi:10.1371/journal.pone.0290153)
Supplement: S1 Table — (DOCX) [file pone.0290153.s004.docx]

**S1 Table. Results of the Dunnet-test in subtests of the HAST.**

|  | **Comparison** | | | | | | | | |
| --- | --- | --- | --- | --- | --- | --- | --- | --- | --- |
|  | **Aphasia–Control** | | | **Aphasia–Stroke** | | | **Control–Stroke** | | |
| **Subtest** | ***Z*** | ***p*** | ***p_adj_*** | ***Z*** | ***p*** | ***p_adj_*** | ***Z*** | ***p*** | ***p_adj_*** |
| Word comprehension | -4.72 | < 0.001 | < 0.001 | -2.73 | 0.01 | 0.01 | 1.28 | 0.20 | 0.20 |
| Sentence comprehension | -7.66 | < 0.001 | < 0.001 | -6.12 | < 0.001 | < 0.001 | 0.32 | 0.75 | 0.75 |
| Repetition | -7.30 | < 0.001 | < 0.001 | -6.13 | < 0.001 | < 0.001 | -0.01 | 0.99 | 0.99 |
| Naming | -6.89 | < 0.001 | < 0.001 | -4.85 | < 0.001 | < 0.001 | 0.96 | 0.34 | 0.34 |
| Word fluency | -8.60 | < 0.001 | < 0.001 | -5.50 | < 0.001 | < 0.001 | 1.79 | 0.07 | 0.07 |
| Total score | -8.50 | < 0.001 | < 0.001 | -5.39 | < 0.001 | < 0.001 | 1.81 | 0.07 | 0.07 |
