# Supplementary material for: The first aphasia screening test in Hungarian: A preliminary study on validity and diagnostic accuracy
Source: PLoS One. 2023 Aug 17;18(8):e0290153. doi: 10.1371/journal.pone.0290153 (PMC10434950; doi:10.1371/journal.pone.0290153)
Supplement: S2 Table — (DOCX) [file pone.0290153.s005.docx]

**S2 Table. Corrected item to total correlations (i.e., item discriminability) and corrected average item to item correlations in the HAST subtests (N = 40).**

|  | **Corrected item to total correlation coefficients (*r*)** | | | |
| --- | --- | --- | --- | --- |
| **Item** | **Word comprehension** | **Sentence comprehension** | **Repetition** | **Naming** |
| **1** | 0.72 | 0.55 | 0.63 | 0.60 |
| **2** | 0.77 | 0.28 | 0.55 | 0.71 |
| **3** | 0.36 | 0.21 | 0.60 | 0.60 |
| **4** | 0.53 | 0.42 | 0.46 | 0.76 |
| **Mean** | 0.60 | 0.37 | 0.56 | 0.67 |
| **Corrected average item to item correlation coefficients (r)** | 0.48 | 0.26 | 0.44 | 0.56 |
