# Supplementary material for: The first aphasia screening test in Hungarian: A preliminary study on validity and diagnostic accuracy
Source: PLoS One. 2023 Aug 17;18(8):e0290153. doi: 10.1371/journal.pone.0290153 (PMC10434950; doi:10.1371/journal.pone.0290153)
Supplement: S3 Table — (DOCX) [file pone.0290153.s006.docx]

**S3 Table. Item passing rates (item difficulty) of the HAST subtests (N = 40).**

| **Item** | **Word comprehension** | **Sentence comprehension** | **Repetition** | **Naming** |
| --- | --- | --- | --- | --- |
| **1** | 0.85 | 0.40 | 0.7 | 0.53 |
| **2** | 0.8 | 0.38 | 0.68 | 0.45 |
| **3** | 0.75 | 0.35 | 0.65 | 0.53 |
| **4** | 0.85 | 0.28 | 0.3 | 0.53 |
| **Mean** | 0.81 | 0.35 | 0.58 | 0.51 |
